# 

**Published:** 1889-10

**Authors:** 


American Public Health Association, Brooklyn, 1889.—The seventeenth annual meeting of this association will be held in the hall of the Brooklyn Institute, Washington and Concord streets, October 22d, 23d, 24th, 25th. Addresses of welcome will be delivered by Hon. Alfred C. Chapin, mayor, on behalf of the city, and by Alexander Hutchins, M. D., on behalf of the medical profession. The following topics have been selected for consideration at the meeting: I. "The Causes and Prevention of Infant Mortality." II. "Railway Sanitation." (*a*) Heating and ventilation of railway passenger coaches; (*b*) Water supply, water-closets, etc.; (*c*) Carrying passengers infected with communicable diseases. III. "Steamship Sanitation." IV. "Methods of Scientific Cooking." V. "Yellow Fever." (*a*) The unprotected avenues through which yellow fever is liable to be brought into the United States; (*b*) The sanitary requirements necessary to render a town or city proof against an epidemic of yellow fever; (*c*) The course to be taken by local health authorities upon the outbreak of yellow fever. VI. "The Prevention and Restriction of Tuberculosis in Man." VII. "Methods of Prevention of Diphtheria, with Results of such Methods." VIII. "How far should Health Authorities be permitted to apply known Preventive Measures for the Control of Diphtheria." IX. "Compulsory Vaccination." X. "Sanitation of Asylums, Prisons, Jails, and other Eleemosynary Institutions." Exhibition of sanitary goods and appliances in another large hall close by.

---

The Southern Surgical and Gynecological Association will hold its second annual meeting in Nashville, Tenn., November 12, 13, and 14, 1889, under the presidency of Hunter McGuire, M. D., LL. D., of Richmond, Va. The preliminary programme is replete with interesting subjects, and reflects great credit upon the efficient Secretary of the Association, Dr. W. E. B. Davis, of Birmingham, Ala. Members of the medical profession are cordially invited to attend, and we have no doubt that a successful and instructive meeting will result.

THE AMERICAN ASSOCIATION OF OBSTETRICIANS AND GYNECOLOGISTS will hold its next annual meeting in Philadelphia, on the third Tuesday of September, 1890, under the presidency of Dr. E. E. Montgomery, of that city.

---

THE AMERICAN GYNECOLOGICAL SOCIETY, at its recent meeting in Boston, voted to hold its next annual meeting in Buffalo.

### BOOKS AND PAMPHLETS RECEIVED.

Essentials of Materia Medica, Therapeutics, and Prescription Writing, arranged in the form of questions and answers, prepared especially for students of medicine. By Henry Morris, M. D. Philadelphia: W. B. Saunders. 1889.

An Introduction to Pathology and Morbid Anatomy. By T. Henry Green, M. D. Philadelphia: Lea Brothers & Co. 1889.

Original Investigations of Cattle Diseases in Nebraska, 1886–1888. By Frank S. Billings, Lincoln, Neb. State Journal Co., printers. 1889.

Transactions of the Mississippi State Medical Association. Twenty-second annual session. Jackson, Miss.: Clarion-Ledger Printing Establishment. 1889.

Index-Catalogue of the Library of the Surgeon-General's Office, United States Army. Volume X. Washington: Government Printing Office. 1889.

Ophthalmology and Ophthalmoscopy, for Practitioners and Students of Medicine. By Dr. Hermann Schmidt-Rimpler. Translated from the third German revised edition. Edited by D. B. St. John Roosa, M. D., LL. D. New York: Wm. Wood & Co. 1889.

A New Operation for the Repair of Lacerated Perineum. By Bernard Burns, M. D. Reprint from Transactions of the American Association of Obstetricians and Gynecologists. 1888.

The Consideration of some Questions Regarding Puerperal Fever. By Charles Warrington Earle, A. M., M. D.

A Few Observations on the Etiology, Prognosis, and Cure of Incipient Cataract without Operative Interference. By A. R. Baker, M. D., Cleveland, Ohio. From Transactions of Ohio State Medical Society. 1889.

Fibromes Uterins; leur Traitement par L'Electrolyse (Method Apostoli) et leur Elmination frequente sous-Muqueuse par L'Action de L'Electricite. Par le Docteur la Torre. Paris: O. Doin, editeur. 1889.

Du Traitement des Fibromes Uterins par la Methode d'Apostoli. Par le Docteur Delatang. Paris: O. Doin, editeur. 1889.

Ophalematoma of the New-Born. By Charles Warrington Earle, M. D. Reprint from the *Journal of the American Medical Association.*

The Treatment of Fractures of the Neck of the Femur by Immediate Reduction and Permanent Fixation. By N. Senn, M. D., Ph. D. Reprint from the *Journal of the American Medical Association.* 1889.

A Year's Experience with Apostoli's Method, with Report of Cases. By A. Lapthorn Smith, B A., M. D. Reprint from the *American Journal of Obstetrics*, August, 1889.

Antiseptic Obstetrics. By Charles Warrington Earle, M. D. Reprint from the Transactions of the Illinois State Medical Society. 1888.

On the Healing of Septic Bone Cavities by Implantation of Antiseptic Decalcified Bone. By N. Senn, M. D., Ph. D. From the *Medical Journal of the Medical Sciences*, September, 1889.

Fatality of Cardiac Injuries. Parts I. and II. By H. A. Hare, M. D. From the *Medical and Surgical Reporter.* 1889.

The Effect of the Entrance of Air into the Circulation. By H. A. Hare, M. D. Reprint from the *Therapeutic Gazette*, September, 1889.

Infant Feeding. By Charles Warrington Earle, M. D. Reprint from the *Journal of the American Medical Association.* 1888.

Cirrhosis of the Pancreas; or, Pancreatic Anemia. By Charles Warrington Earle, M. D. Reprint from the Transactions of the Illinois State Medical Society.

Rapid Dilatation of the Cervix Uteri. Surgical Treatment for Lacerations of the Perineum and Pelvic Floor. A Successful Vaginal Hysterectomy for Carcinoma Uteri. The Pathology of Ectopic Pregnancy and Pelvic Hematocele. By Wm. H. Wathen, M. D. 1889.

The Influence of Sewerage and Water Pollution on the Prevalence and Severity of Diphtheria. By Charles Warrington Earle, M. D. Reprint from the *Archives of Pediatrics*, November, 1888.

Observations in Vienna. The General Hospital, Billroth, Carl Braun, Bandl, and others. By Charles Warrington Earle, M. D. Reprint from the *Western Medical Reporter.* 1888.

The Treatment (not Preventive) of Puerperal Fever. By Charles Warrington Earle, M. D. From the Chicago *Medical Reporter and Examiner.*

Address of the President, C. W. Earle, delivered at the thirty-ninth annual meeting of the Illinois State Medical Society, May 21, 1889. Responsibilities and Duties of the Medical Profession Regarding Alcoholic and Opium Inebriety.

State Board of Health of New York. Local Health Service of the State. Extract from Ninth Annual Report.

Cornell University, College of Agriculture. Bulletin of the Agricultural Experiment Station, Horticultural Department. IX. September, 1889. A Study of Windbrakes in their Relation to Fruit-Growing. Published by the University. 1889.

McGill University. Annual Calendar, Faculty of Medicine. Montreal. 1889.

New York Post-Graduate Medical School and Hospital. Eighth Annual Announcement, Session of 1889–'90.

Western Reserve University, Medical Department, Cleveland, Ohio. Annual Announcement, Session 1889–'90.

University of Maryland. Eighty-third Annual Circular of the School of Medicine, Session 1889–'90. Baltimore, Md.

Thirteenth Annual Announcement of Ensworth Medical College and Hospital, St. Joseph, Mo. Session 1889–'90.

State Board of Health Bulletin. Nashville, Tenn., August 20, 1889.

Monthly Bulletin of the New York State Board of Health, July, 1889.

Health in Michigan, August, 1889.

Weekly Abstract of Sanitary Reports. Volume IV., Nos. 35, 36, 37, 38.

---

### Literary and Other Notes.

### PROGRESS OF INVENTIONS SINCE 1845.

IN THE year 1845, the present owners of the *Scientific American* newspaper commenced its publication, and soon after established a bureau for the procuring of patents for inventions at home and in foreign countries. During the year 1845 there were only 502 patents issued from the United States Patent Office, and the total issue from the establishment of the Patent Office, up to the end of that year, numbered only 4,347.

Up to the first of July this year there have been granted 406,413; showing that since the commencement of the publication of the *Scientific American* there have been issued from the United States Patent Office 402,166 patents, and about one-third more applications have been made than have been granted, showing the ingenuity of our people to be phenomenal, and much greater than ever the enormous number of patents issued indicates. Probably a good many of our readers have had business transacted through the offices of the *Scientific American,* in New York or Washington, and are familiar with Munn & Co.'s mode of doing business, but those who have not will be interested in knowing something about this, the oldest patent soliciting firm in the country, probably in the world.

Persons visiting the office of the *Scientific American*, 361 Broadway, New York, for the first time, will be surprised, on entering the main office, to find such an extensive and elegantly equipped establishment, with its walnut counters, desks, and chairs to correspond, and its enormous safes, and such a large number of draughtsmen, specification writers, and clerks, all busy as bees, reminding one of a large banking or insurance office, with its hundred employees.

In conversation with one of the firm, who had commenced the business of soliciting patents in connection with the publication of the *Scientific American*, more than forty years ago, we learned that his firm had made application for patents for upward of one hundred thousand inventors in the United States, and several thousand in different foreign countries, and had filed as many cases in the Patent Office in a single month as there were patents issued during the entire first year of their business career. This gentleman had seen the Patent Office grow from a sapling to a sturdy oak, and he modestly hinted that many thought that the *Scientific American*, with its large circulation, had performed no mean share in stimulating inventions and advancing the interests of the Patent Office. But it is not alone the patent soliciting that occupies the attention of the one hundred persons employed by Munn & Co., but a large number are engaged on the four publications issued weekly and monthly from their office, 361 Broadway, New York, viz.: The *Scientific American*, the *Scieutific American Supplement*, the Export Edition of the *Scientific American*, and the Architect and Builders' Edition of the *Scientific American*. The first two publications are issued every week, and the latter two the first ot every month.

---

Kilmer's Physician's Pocket Ledger.—We have been using this unique and useful account-book nearly a year, and regard it as time-saving and practical. We, therefore, gladly subjoin the following testimonials:

It is the only perfect ledger I ever saw, and no physician should think of buying any other. W. L. REED, M. D.,
Professor of Materia Medica, St. Louis (Mo.) Medical College.

I would not do without it. It benefits me $500 a year in making collections alone. E. W. McALLISTER, M. D.,
South Bend, Ind.

For descriptive circulars, address Dr. S. S. Kilmer, South Bend, Ind.

